# Repeated hypoxic episodes allow hematological and physiological habituation in rainbow trout

**DOI:** 10.3389/fphys.2024.1289903

**Published:** 2024-02-08

**Authors:** Nuria Ruiz, Irene García-Meilán, Ali Reza Khansari, Mariana Teles, Josep Pastor, Lluís Tort

**Affiliations:** ^1^ Department of Cell Biology, Physiology and Immunology, Universitat Autònoma de Barcelona, Barcelona, Spain; ^2^ Department of Cell Biology, Physiology and Immunology, Universitat de Barcelona, Barcelona, Spain; ^3^ Departament of Animal Medicine and Surgery, Universitat Autònoma de Barcelona, Barcelona, Spain

**Keywords:** repeated stress, hematology, fish, rainbow trout, hypoxia, dissolved oxygen concentration, cortisol

## Abstract

**Introduction:** Under climate change, the increase in temperature in aquatic environments may induce oxygen depletion. In extreme cases, low oxygen may become a limiting factor for fish, thus generating stress. In addition, consecutive hypoxic episodes may complicate the recovery of individuals and hinder their ability to modulate physiological and biochemical responses to maintain homeostasis. Thus, the aim of this study was to determine the hematological and physiological responses of rainbow trout under a condition of repeated hypoxic and manipulation stresses at three different time points.

**Methods:** Every hypoxic episode consisted of exposing the fish to low dissolved oxygen concentrations (2 mgO_2_/L for 1 h). Following the exposure, the fish were allowed to recover for 1 h, after which they were sampled to investigate hematological and physiological parameters.

**Results and discussion:** The results showed a pattern of habituation reflected by values of hematocrit, hemoglobin, and mean corpuscular volume, indicating a certain ability of rainbow trout to resist this type of repeated hypoxic events, provided that the fish can have some recovery time between the exposures.

## 1 Introduction

The current context of climate change is expected to strongly affect fish because of its consequences on water temperature and other tightly linked quality parameters. The expected changes are related to biotic (primary production, pathogens, and food availability) and abiotic (temperature, salinity, pH, and, particularly, hypoxia) factors, affecting aquatic organisms in their distribution, growth, size, and overall health (Action, 2020). As wild populations may be exposed to more than one of these environmental stressors, much uncertainty is generated about the consequences of these stressors and their potential synergy (e.g., hypoxia and temperature rise) (Petitjean et al., 2019). As fish are aerobic organisms, the concentration of dissolved oxygen (DO) is a limiting factor both in the environment and in production systems, and its availability depends on its amount and solubility. Episodes of repeated hypoxia may become more common in the future. For instance, diel cycling hypoxia, a worldwide phenomenon affecting freshwater and coastal systems due to an increase in partial pressure during daylight hours associated with photosynthetic activity and a subsequent diminution overnight as a consequence of biological demand, is particularly common during spring and summer months (Williams et al., 2019). In natural environments, it can be a result of eutrophication and increasing temperatures. Furthermore, hypoxic phenomena may be amplified in aquaculture settings because of overstocking or overfeeding (Bera et al., 2017).

Hypoxia occurs when oxygen concentrations drop low enough to cause negative physiological, immune, and behavioral effects on fish, affecting their growth and overall performance (Abdel-Tawwab et al., 2019). This can affect acutely or chronically, depending on the duration and recurrence of the impact of temperature change, seasonality, water flow, and/or chemical composition (Xiao, 2015). Most species have a high ability to habituate to fluctuating oxygen levels, modifying O_2_ uptake, delivery, and metabolism to prevent hypoxemia and energy exhaustion (Boutilier, 2001). However, if fluctuations are recurrent, they can compromise the overall health and lead to increased mortality. Nevertheless, preconditioning exposure to hypoxia demonstrated the existence of physiological plasticity in fish (Borowiec et al., 2015), even in rainbow trout, which is considered a species particularly sensitive to this stressor (Gamperl et al., 2001). Therefore, to maintain optimal fish performance, growth, and feeding, oxygen levels should be kept near saturation. Ideally, DO levels in aquaculture settings should be above 5 mgO_2_/L, which is the accepted baseline for most species (Abdel-Tawwab et al., 2019). Although under aquaculture conditions, DO levels are generally monitored and controlled, some factors, such as overcrowding or abnormal increases in temperature, may lead to suboptimal rearing conditions, and if the fish are unable to adapt, they may develop a stress response and maladaptation (Magnoni et al., 2019).

The stress response is a very primitive and highly conserved response in extant species since it preserves the organism’s homeostasis (Barton, 2002). By activating primary, secondary, and tertiary responses (Schreck and Tort, 2016), it has been shown that animals are able to adapt to different stressors, including hypoxia, by reducing their physiological costs and modifying behavioral responses (Conde-Sieira et al., 2018; Alfonso et al., 2020). However, the capacity for habituation will depend on several factors, namely, the species itself, as well as the type and intensity of stress (Nilsson et al., 2012; Koakoski et al., 2013). Moreover, as the importance of animal welfare and its public awareness in production systems is increasing, the maintenance of suitable conditions for fish husbandry should consider the consequences of these stress episodes.

In the present work, we monitored the hematological and physiological parameters as they can be suitable indicators of the process of oxygen uptake and distribution to tissues (Fazio, 2019). The number of red blood cells (RBCs), hemoglobin concentration (HGB), hematocrit (HCT), mean corpuscular volume (MCV) of red blood cells, and mean hemoglobin concentration (MHC) are most commonly used to evaluate the hematological status in fish (Pavlidis et al., 2007). However, other less-investigated factors, such as the widening of the distribution of red blood cells (RDW), a parameter related to the pathway of ionic erythropoiesis to measure the size and volume of erythrocytes, have been monitored as additional indicators of health status, as shown in humans (Fish et al., 2019). In addition, the determination of the number of white blood cells (WBCs) provides information on the animal’s immune status, as well as the proportion of heterophilic cells (neutrophils, basophils, and eosinophils) and mononuclear cells (lymphocytes and monocytes). Finally, the number of platelets (PLTs) was also assessed (Fazio, 2019). Altogether, the correlation between hematological parameters, as indicators of oxygen uptake and distribution (Abdel-Tawwab et al., 2019), and physiological stress markers, such as cortisol, glucose, and lactate in plasma (Groff and Zinkl, 1999), provides valuable insights on the specific physiological compartments of functional allostasis, thus becoming markers of a range of physiological variations and useful tools to detect, identify, and calibrate specific stressors.

Many studies have addressed the hypoxia effects on fish (Pollock et al., 2007; Xiao, 2015), particularly in rainbow trout (*Oncorhynchus mykiss*) (see, for instance, the work of Pilgaard et al. (1994), Sappal et al. (2016), and Zhang et al. (2018)). The present study focuses on two aspects that have been discussed in previously published work. First, studies on hypoxia have dealt with fish that have been taken out of the water, so, effectively, subjected to anoxia. Furthermore, these fish are simultaneously subjected to the consequent handling stress associated with catching and restricting the fish out of the water. However, these two stressors are difficult to separate. Second, studies on repeated hypoxia are scarce, and the ability of fish to respond repeatedly to this stressor is, therefore, less understood. Since hypoxic episodes may become more frequent in the context of climate change, the aim of the present research was to determine the effects of repeated hypoxia and manipulation by subjecting the animals to one, two, or three acute hypoxic shocks and to determine whether there is a cumulative effect, a certain adaptive capacity or, on the contrary, a constant response. In addition, the stress recovery phases, specifically at 1, 6, and 24 h after hypoxia and manipulation, were also investigated.

## 2 Materials and methods

### 2.1 Fish and rearing conditions

A total of 135 rainbow trout (*Oncorhynchus mykiss*) with a mean weight of 62.89 ± 11.10 g, a mean length of 17.58 ± 1.09 cm, and a condition factor of 1.2 ± 0.1 were obtained from a local fish farm (Molinou, Rialb, Spain) and acclimated to a recirculating aquaculture system (RAS) in the facilities of the Autonomous University of Barcelona (AQUAB) for 2 weeks. The RAS is equipped with water pumps, a recirculating cooling system, a sand filter, a biofilter, and an aeration system for the tanks, allowing for the DO concentrations to be maintained between 7.20–8.10 mgO_2_/L. The photoperiod was held at 12 L: 12 D, and an average temperature of 14.6°C ± 0.3°C was maintained. Throughout the acclimation and experimental periods, the density was 6.80 ± 0.1 Kg/m^3^. All experimental procedures involving fish were submitted and authorized by the Ethics and Animal Care Committee of the “Universitat Autònoma de Barcelona” (permit numbers OH4218 4219 and DAMM 11251), in accordance with the international Guiding Principles for Biomedical Research Involving Animals (EU2010/63).

### 2.2 Experimental design

Five experimental groups were established, divided into two control groups and three treatment groups, each undergoing a different number of manipulation and hypoxic shocks. Each group had 9 fish per sampling time, with a total of 27 rainbow trout per group. The first group was the absolute control (AC) group, being the only group that was sampled without prior manipulation. Since it is known that the manipulation of individuals is an additional stressor to hypoxia, a manipulated control (MC) group was considered with the aim of determining how manipulation by itself can alter the evaluated parameters without decreasing the DO concentration. The remaining three groups were subjected to hypoxic shocks, all handled in the same manner as the MC group and subsequently exposed to a decreased concentration of oxygen. The H1 group was exposed to hypoxic conditions once, whereas fish in the H2 group were challenged with two hypoxic shocks, and the H3 group was subjected to low levels of DO for a total of three hypoxic shocks. Thus, the H2 and H3 groups suffered repeated acute hypoxia stress, with 48 h between hypoxia shocks. The MC group is only comparable to the H1 group, as H2 and H3 had more manipulations than the MC group. Since H2 and H3 were only compared with the AC group, we cannot exclude the effect of hypoxia and manipulation in these groups. In the groups where several hypoxic shocks were performed, the fish were returned to the acclimation tank under normal oxygen and water physicochemical levels until the next shock. Once the fish experienced the last shock, they were transferred to the recovery tank until sampling. As the number of fish in the tank decreased after each sampling, the water volume was accordingly decreased with the aim to maintain the density at 6.80 ± 0.1 Kg/m^3^.

### 2.3 Hypoxic shock and the sampling procedure

**FIGURE 1:**
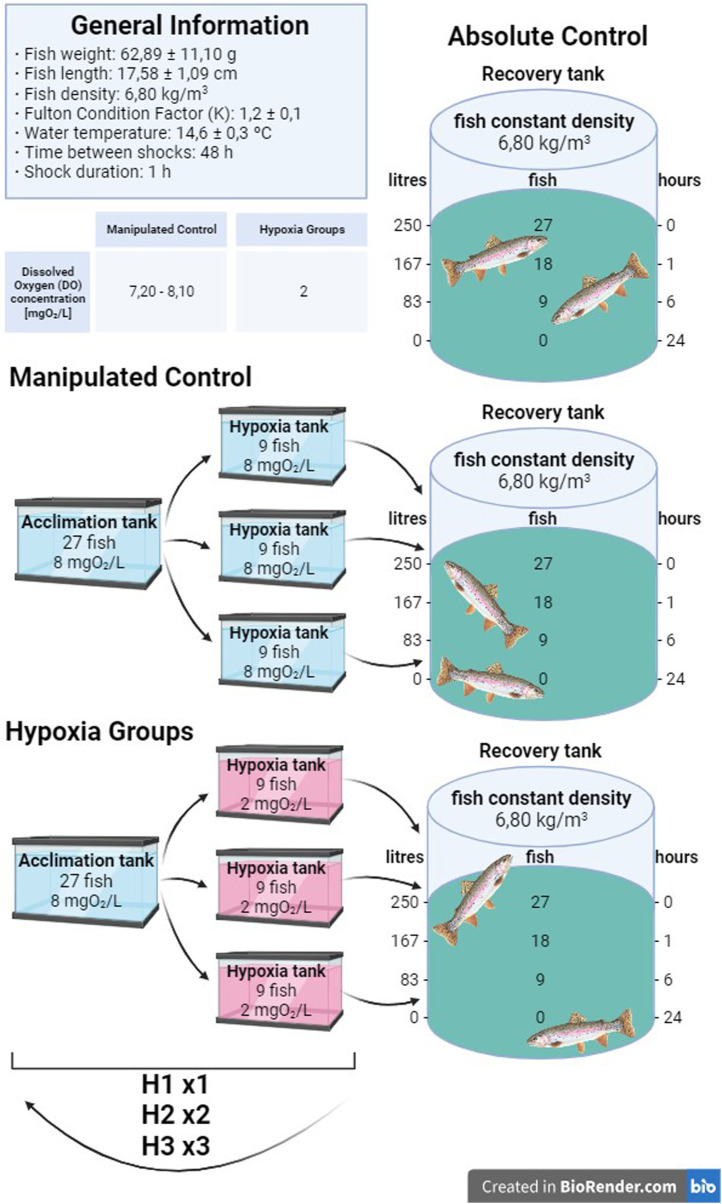
Graphical abstract of the design of the experiments.

Hypoxic levels were reached by decreasing the DO concentration by adding nitrogen gas (N_2_) into the water (i.e., 5 min to reach hypoxic condition), as in the work of Schurmann and Steffensen (1992), and not removing the fish from the water. Three tanks were used to replicate the treatment procedure by maintaining a density of 6.80 ± 0.1 Kg/m^3^. After the procedure, all fish were placed in the same recovery tank to avoid any tank effect. The shock included catching the fish from the experimental tanks and putting them into the hypoxia tanks, where the DO concentration was decreased from 7.5 ± 0.5 to 2.2 ± 0.5 mgO_2_/L by adding nitrogen gas. The fish were introduced once the oxygen level had reached the desired values and remained there for 1 h. It should be added that during the shock, the DO concentration was continuously monitored to check that the levels were within the study range and to avoid a decrease below 1.5 mgO_2_/L, which would prove lethal to this species. Throughout the experiment, the density was constantly maintained at 6.80 ± 0.1 Kg/m^3^ (Figure 1). Following the hypoxia shock, the fish were transferred to a recovery tank with 7.2—8.1 mgO_2_/L, which was isolated from the system to avoid any possible circulating cortisol levels affecting the other fish, as a chemical detection by peripheral cortisol receptors that may affect fish response has been previously suggested (Kolosov and Kelly, 2019). After the last hypoxia shock, the fish were transferred to a recovery tank where sampling took place. As the number of fish per tank changed, the water volume was adjusted to maintain the same density throughout the experiment. By the time the sampling of the experimental group was finished, the oxygen and water turnover levels were returned to optimal levels. For sampling, a total of 27 fish per group, with 9 fish per time point of the hypoxia and treatment groups (AC, MC, H1, H2, and H3), were anesthetized with sublethal doses of tricaine methanesulfonate (MS-222) buffered with sodium bicarbonate. All fish were weighed and measured, and blood samples were collected within 3 min. After that, fish were sacrificed by sectioning the spinal cord.

### 2.4 Hematological analysis

Blood collection was performed using a heparinized syringe through caudal puncture. The first 500 *μ*L aliquot of blood was added to Eppendorf tubes containing heparin (1:40) for the determination of hematological parameters. A second aliquot was centrifuged at 1500 *g* for 10 min to collect the plasma, which was subsequently frozen at −20°C until the physiological analysis. Hematological analyses were performed within 2–12 h after blood sampling using the automated flow cytometer blood cell analyzer Sysmex XN-1000V for veterinary use (Sysmex Corporation, Kobe, Japan). Internal quality control (QC) was performed daily using three levels of commercially available QC material (Sysmex XN Check level 1 or low range, level 2 or normal range, and level 3 or abnormal high range; Sysmex Corporation, Kobe, Japan).

### 2.5 Physiological analysis

For cortisol analysis, the plasma was first diluted in the analysis buffer, and the aliquots were frozen at -20°C for at least 24 h until analysis with an ELISA test using a commercial EIA kit (Cortisol ELISA Kit; Neogen^®^ Corporation, Ayr, United Kingdom) following the manufacturer’s instructions. This kit has been previously validated for this species and used in past experiments (Carbajal et al., 2019).

Glucose and lactate analyses were performed using colorimetric test kits (LO-POD glucose and LO-POD lactate, SPINREACT, Spain) following the manufacturer’s recommendations.

### 2.6 Statistical analysis

The obtained data were analyzed with a generalized linear model (GzLM) using the Gaussian family for all the variables using RStudio (R Core Team 2022. R: a language and environment for statistical computing, R Foundation for Statistical Computing, Vienna, Austria, URL https://www.R-project.org/). Data normality and residual distribution of the model were checked through Shapiro–Wilk tests. The factors analyzed were hypoxia treatments, time, and the interaction between them. If significant differences (*p*-value < 0.05) were found, pairwise comparisons by Tukey’s correction were applied. Therefore, variables where only the effect of treatment is found are presented first, followed by those with only time effect significance, and finally, all the parameters that had interaction between the two factors. For the pairwise comparisons, the effects of manipulation and hypoxia were differentiated and, therefore, AC, MC, and H1 were compared on one side and AC, H1, H2, and H3 on the other. Regarding treatment and time effect, it has to be added that only when one factor was significant, all the data were gathered by treatment or time, depending on the case.

## 3 Results

In this section, the results are presented according to the significant effect of each factor and the significant interaction between them in the order mentioned above. As can be observed in the figures, the significance is shown for the differences among the factors. For instance, a parameter included only in the treatment effect indicates that significant differences can be found between treatments but not between times or with interacting factors.

### 3.1 Treatment effects

The number of RBCs, the amount of HGB and PLTs, and MHC were affected by treatment (Table 1) but not by time or interaction between them. An increase in RBC and HGB levels, likely due to handling stress, was observed in the MC compared to the AC group, whereas one hypoxic shock tended to decrease these values since the values of H1 are lower than MC and, in theory, it had to be higher due to manipulation. In addition, significantly higher RBC values were measured in H2 compared to H1, with H3 displaying intermediate values, similar to those found in AC.

**TABLE 1:** RBC number, HGB, PLT number, MHC, and heterophil cells in percentage in rainbow trout. Data are represented as mean ± SEM (*n* = 27 fish per treatment). Significant differences between treatments are marked with different letters (*p*-value < 0.05). AC, absolute control; MC, manipulated control; H1, one hypoxic shock; H2, two hypoxic shocks; and H3, three hypoxic shocks.

Group	RBC (10^3^/*μ*L)	HGB (g/dL)	PLT (10^3^/*μ*L)	MHC (pg)	Heterophils (%)
AC	0.961 ± 0.0224 ^*b*^	5.57 ± 0.108 ^*ab*^	2.34 ± 0.140	50.8 ± 0.770 ^*ab*^	17.5 ± 1.37 ^*b*^
MC	1.058 ± 0.0229 ^*a*^	5.78 ± 0.110 ^*a*^	2.34 ± 0.162	48.3 ± 0.770 ^*bc*^	20.1 ± 1.40^*b*^
H1	0.939 ± 0.0224 ^*b*^	5.61 ± 0.108 ^*ab*^	2.38 ± 0.149	52.8 ± 0.770 ^*a*^	22.0 ± 1.49 ^*ab*^
H2	1.031 ± 0.0229 ^*a*^	5.77 ± 0.123 ^*ab*^	2.28 ± 0.154	49.5 ± 0.846 ^*bc*^	27.3 ± 1.46 ^*a*^
H3	0.975 ± 0.0224 ^*ab*^	5.35 ± 0.108 ^*b*^	2.61 ± 0.154	47.6 ± 0.770 ^*c*^	19.3 ± 1.43 ^*b*^

PLT count displayed a decreasing trend with an increase in the number of hypoxic shocks and manipulations, although above the threshold for statistical significance (*p* < 0.07; Table 1), except for H3, which showed increased PLT values. Finally, the results indicated an increase in MHC in H1 compared to MC, with a return to AC in H2, and a significant reduction in MHC in H3 compared to AC (Table 1). Furthermore, the results suggest that both hypoxia plus manipulation and time independently alter the proportion of heterophilic and mononuclear cells, although no interactive effect of these factors was found. It should be noted that H2 is the treatment group that showed the highest levels of heterophils compared to the AC and H1 groups, regardless of the time after hypoxia exposure and manipulation (Table 1).

### 3.2 Time effects

**FIGURE 2:**
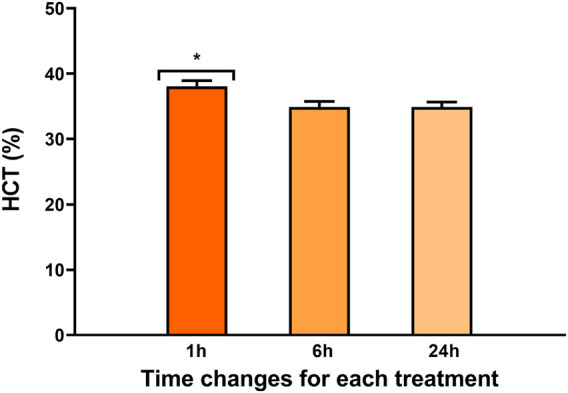
Percentage of hematocrit in rainbow trout blood 1, 6, and 24 h after treatment. Data are represented as mean ± SEM (*n* = 45 per sampling time). The significant differences in sampling time are indicated by an asterisk (*p*-value < 0.05).

**FIGURE 3:**
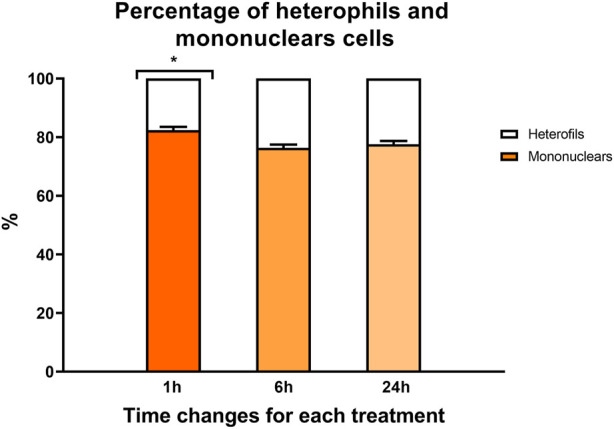
Percentage of mononuclear cells (color bars) and heterophils (white bars) in rainbow trout blood 1, 6, and 24 h after treatment. Data are represented as mean ± SEM (*n* = 45 per sampling time). The significant differences in sampling time are indicated by an asterisk (*p*-value < 0.05).

A time effect was observed in both hematocrit values (Figure 2) and the percentage of heterophilic and mononuclear cells (Figure 3). Interestingly, in both parameters, a significant increase was observed at 1 h regardless of the experimental treatment.

### 3.3 Interaction effects

**FIGURE 4:**
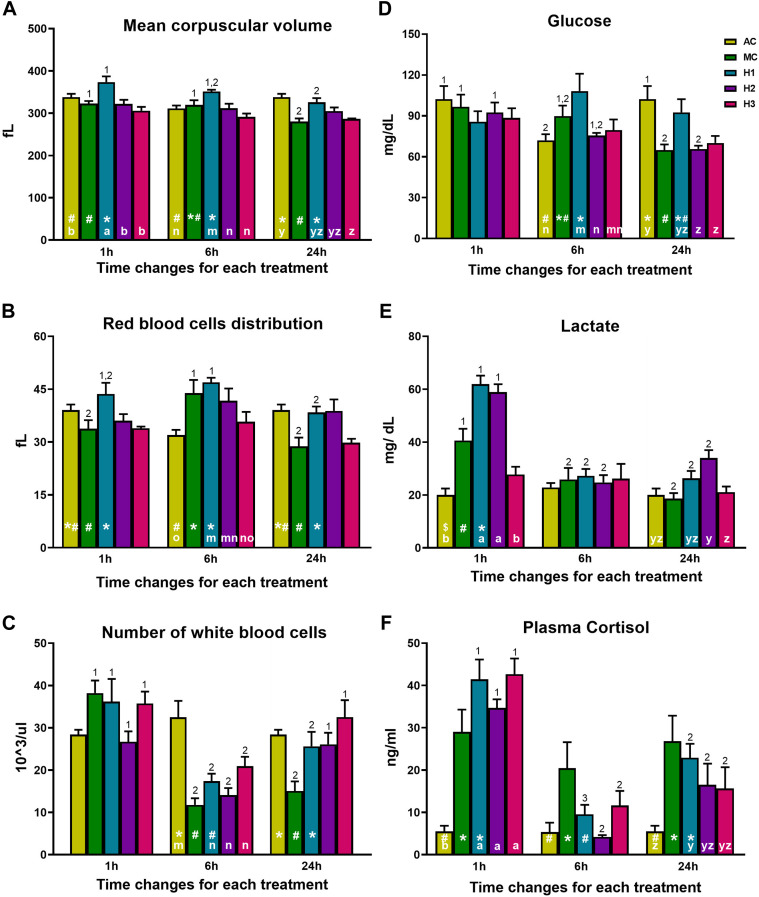
**(A)** Mean corpuscular volume, **(B)** erythrocyte distribution width (RDW-SD), **(C)** number of white blood cells, **(D)** plasma glucose, **(E)** plasma lactate, and **(F)** plasma cortisol in rainbow trout blood 1, 6, and 24 h after treatment. Data are represented as mean ± SEM (*n* = 9 per sampling time). Significant differences (*p* < 0.05) between treatments at the same sampling time are shown with different letters (a/b at 1 h, m/n at 6 h, and y/z at 24 h after treatment). Significant differences in sampling time among the same treatment are shown by numbers (*p*-value < 0.05). Differences between AC, MC, and H1 are shown by * and #.

A significant interaction between manipulation and exposure to hypoxia and time was found in MCV, RDW, WBC, glucose, lactate, and cortisol (Figure 4). In H1 and MC groups, a gradual reduction of the MCV can be observed over time t = 1 h vs. 24 h. In addition, H1 also presented higher MCV levels at 1 and 6 h compared to both AC and MC and the other groups that underwent manipulations and hypoxic shocks (H2 and H3) (Figure 4A). RDW showed differences between AC, MC, and H1 in all the sampling times and AC with all the groups that underwent manipulation and hypoxic exposures only at the sampling point t = 6 h. On one side, looking at t = 6 h, it can be observed that H1 and MC are the groups with the highest values, and AC is the group with the lowest values. On the other side, at t = 6 h, comparing the groups that underwent manipulations and hypoxia, H1 is the group with the highest values. In addition, the more the manipulations and hypoxias, the less the RDW values. Thus, H3 shows similar values as AC. The most relevant difference regarding time occurs in MC and H1 groups, showing lower RDW values at 1 h and 24 h after treatment than at 6 h. This trend was also found when comparing all experimental groups versus AC, being higher in H1 and, therefore, suggesting a greater anisocytosis 6 h after treatment (Figure 4B). Regarding immune cells, the lowest WBC levels occurred 6 h after either manipulation or hypoxic exposure when compared with AC. In MC, these differences are maintained even after 24 h; however, this is not observed in the groups subjected to repeated hypoxia and manipulations (H2 and H3) (Figure 4C). In terms of physiological response, plasma glucose levels significantly increased 6 h after treatment in H1 compared with AC and all the groups that suffer manipulation and hypoxia. In contrast, glucose values were reduced at 24 h, except in the MC and H1 groups (Figure 4D). Lactate levels clearly increased 1 h after exposure and then decreased until recovering basal levels, except for the H2 group, which presented higher levels at 24 h than AC. It should be highlighted that lactate did not peak at 1 h in the H3 group, as observed in H1 and H2 (Figure 4E). Finally, a significant rise in plasma cortisol levels at 1 h post-exposure was observed in rainbow trout that underwent hypoxic shock and manipulation (H1, H2, and H3) compared with the AC group. Similarly, the MC group also had elevated cortisol levels when compared to the AC group. The cortisol levels dropped at 6 h and then interestingly rose again at 24 h (Figure 4F). Significant differences were maintained at 24 h post-shock between AC and both MC and H1, but not H2 nor H3, both groups showing intermediate values.

## 4 Discussion

In this work, the results are presented in two sets of groups, the first including the control with the manipulated control and one hypoxia exposure groups and the second including the groups experiencing repeated manipulations and hypoxias. In the first set, AC represents the basal levels that are expected to represent the basal resting values. The MC group allows to differentiate between the basal and manipulated fish, and finally, hypoxia 1 (H1) allows to differentiate the effects between the manipulated and one hypoxia groups. In the second set, we compare the effect of repeated manipulations and hypoxia exposures related to AC as the basal levels of our animals. Since there is no MC group with two and three manipulations, we excluded the MC group from the analysis of this second set of groups as they are not directly comparable. The overall results suggest that rainbow trout are able to cope with repeated manipulation and acute hypoxia (1 h with DO levels down to 2 mgO_2_/L) and recover from the shock 24 h after the stressor and that subjecting the fish again to the same stressor leads to a certain level of habituation. Among the hematological variables, red blood cells and hemoglobin showed an increase in the MC group, presumably due to the handling of the animals, as observed in the work of Acerete et al. (2004). On the contrary, as observed in H1, hypoxia generates a decrease in RBC, probably due to an excessive alteration of the oxygen delivery system, resulting in an reduction in the overall metabolism and activity (Pichavant et al., 2002). After the second manipulation and shock (H2), the RBC level increased, suggesting a recovery in oxygen content and an improvement in blood transport (Aboagye and Allen, 2018), and recovered control values in H3. A similar trend was observed in HCT, which tended to diminish as exposures and manipulations increased. Moreover, the increase of HCT 1 h after manipulation and/or treatment could be associated with balancing out the additional requirements for oxygen under hypoxia (Muusze et al., 1998). All these data suggest an adaptation of the animals, resulting in a lower use of oxygen in habituated fish (Petersen and Gamperl, 2011; Remen et al., 2012).

Significant differences in MCV were observed both between groups and sampling points. The highest MCV was observed in H1, which indicates that the erythrocyte volume increased. This could be explained by two mechanisms that have been previously described. On one hand, the osmoregulatory changes that occur over time in an attempt to increase the efficiency of oxygen transfer through the cell membrane (Aboagye and Allen, 2018). On the other hand, the increase in HCT and MCV values, without much change in RBC, may suggest the swelling of erythrocytes (Aboagye and Allen, 2018). The swelling of the erythrocytes has been observed as a result of catecholamine release after stress, trying to compensate for the efficiency of cell oxygen uptake due to the increased demand of oxygen, in this case caused by the hypoxic stress and manipulations (Caldwell and Hinshaw, 1994). Interestingly, both HCT and MCV displayed a correlation with MHC. In this case, the groups with higher levels of HCT and MCV presented higher MHC. This could be explained by cellular hemolysis, which may occur during the first hour after the manipulation and hypoxic shock, as previously demonstrated (Caldwell and Hinshaw, 1994; Muusze et al., 1998). Furthermore, this effect is reduced over time, suggesting recovery. This idea of hemolysis was initially proposed by Swift and Lloyd (1974), describing how HCT and MCV values increase because of cell swelling, which causes hemolysis. Afterward, differences were also observed between treatments, and as in the rest of the erythrocyte-related parameters, a certain degree of habituation can be observed in the groups subjected to hypoxia. Therefore, the more the hypoxia shocks accumulate, the lower the response, as previously seen during chronic hypoxia exposure (Caldwell and Hinshaw, 1994).

It has been previously demonstrated that stressors, such as infections by trematode metacercaria, entail a significant decrease in the distribution by the size and volume of the cell (i.e., RDW) (Khan et al., 2017). In other studies associated with nutritional stress, an inverse relationship between RDW and other hematological parameters, such as MCV or HGB, was observed (Goran et al., 2017). In the present study, RDW resulted higher 6 h post-shock, suggesting greater heterogeneity and, therefore, greater anisocytosis, since some cells are recovering while others are not, thus increasing the heterogeneity levels at this time point. After 24 h, homogeneity is restored, but since the cells are smaller because no swelling is produced, the values at 24 h are lower than those at 1 h. It should be added that this parameter has been little studied in fish; however, in humans, a decrease in RDW levels may reflect anemia, implying that erythrocytes do not have the capacity to efficiently transport oxygen (Maiolo et al., 1991). As it has been seen with the rest of the parameters, in the case of H3, RDW returns to the levels of the AC at 1 and 24 h, suggesting, once again, habituation. For white blood cells, all treatments showed a decrease at 6 h with respect to the absolute control group, especially fish subjected to two hypoxic shocks that also presented a significant decrease at 1 h after treatment. These results agree with those found by Sheng et al. (2019) in the face of repeated stress (hypoxia and manipulation) and may be related to cell damage detection, one of the functions of WBCs (Sheng et al., 2019; Missinhoun et al., 2021). Moreover, H3 rainbow trout showed an ability to recover WBC levels, further suggesting habituation.

Among white blood cells, two different cell types can be distinguished depending on their nuclear morphology: heterophils and mononuclear cells. Heterophils can be further classified into neutrophils, eosinophils, and basophils. The majority of heterophils are neutrophils, which do not show relevant changes when there is circulating cortisol, as described by McLeay (1973). On the other hand, in the face of a stressful situation, eosinophils have been shown to decrease, whereas no changes have been observed in the basophil populations. In this sense, the depletion of eosinophils would match with the peak of plasma cortisol (Wojtaszek et al., 2002). Similarly, mononuclear cells can be subdivided into monocytes and lymphocytes. These authors demonstrated a lack of changes in monocyte levels when facing a stressful situation, whereas lymphocytes displayed a significant reduction in their abundance. The decrease in monocytes observed in the present study could be explained by the combination of the reduction of lymphocytes and the small proportion of eosinophils among this group of cells. Further studies are needed to ascertain the less-known consequences of hypoxia and manipulation on white blood cells.

The main function of PLTs is related to clotting, and since no lesions were observed at any point of the experiment, no differences were expected in levels of these particular cells after hypoxia and manipulation. In addition, it has been reported that cortisol levels do not alter thrombocyte levels (Wojtaszek et al., 2002). Although a trend of decrease by repeated hypoxia and manipulation is suggested, differences were not significant.

The variation observed in cortisol levels between treated groups and control ones is commonly found in stress studies and is particularly associated with handling procedures (Acerete et al., 2004; Sadoul and Geffroy, 2019). This may also be the case in our results under hypoxia exposure since, as observed in the work of O’Connor et al. (2011), neither acute nor chronic exposure to hypoxia caused significant differences with respect to the control groups. On the other hand, handling is a stressor to which the fish responds with increased cortisol levels, although the potential for habituation has been suggested (Pagès et al., 1995), so manipulation and hypoxia can raise the levels of cortisol, as observed in this study. In addition, it can be observed that the WBC levels seem to mimic the rise in cortisol levels and, therefore, the activated response of the hypothalamic–pituitary–interrenal axis (Pottinger and Carrick, 1999), as cortisol and WBC levels show a high correlation (Figures 4C, F). Similar results have been observed in other studies, such as in the work of Carbajal et al. (2019), where WBCs were higher in a polluted environment under suboptimal conditions inducing neutrophilia and/or lymphopenia in fish. Furthermore, the increase in WBCs is related to the migration of these cells from the spleen to the blood (Barcellos et al., 2004). Moreover, regarding cortisol measurements, two different types of response patterns can be observed. On one hand, the groups that were not subjected to hypoxia did not display any significant differences between sampling points, although statistically significant differences arise when comparing the AC and MC groups, as expected. On the other hand, there is a significant reduction in cortisol levels in all groups exposed to hypoxic conditions at t = 6h, with a subsequent significant increase at t = 24 h. This could be partly due to circadian rhythms inherent to the species, as described by Naderi et al. (2018), although the lack of differences within the AC and MC groups suggests there are other factors at play. Therefore, it can be hypothesized that the decrease observed at t = 6 h reflects the start of the recovery period but that the fish have developed, to some extent, a predictable behavior to this specific stressor, as both shocks occurred at the exact same time 1 day apart. This would explain the significant increase in cortisol levels at t = 24 h in the treatment groups. Nonetheless, further research is needed to investigate this possible adaptive behavior in *O. mykiss*. This hypothesis is supported by the fact that the predictability in fish was demonstrated after a positive stress stimulus (i.e., feeding) or a negative one (i.e., crowding) by changes in plasma cortisol levels (Galhardo and Oliveira, 2009; Galhardo et al., 2011), and the anticipatory responses are not unusual in animals. This fact allows self-preparation for the upcoming event, giving to the fish a certain control capacity on the stressor response, thus optimizing the efficacy of the response (Galhardo et al., 2011). In our work, plasma cortisol levels 24 h after the hypoxic shock were higher in hypoxic groups versus the AC group, suggesting that there is no lower response as expected. This difference should be attributed to the type of the stressor, which, in this case, is hypoxia in water instead of anoxia, like in most of the studies. We must consider that the predictability response depends on the stressor properties (nature, intensity, and frequency), the signaling cascade, and the time elapsed between the signal and the onset of the negative event, as it is currently observed in stress studies (Galhardo and Oliveira, 2009).

At this point, it is interesting to emphasize that, in the present work, hypoxia was achieved by reducing the oxygen concentration in water, which is rather different than the hypoxia caused by air exposure. The latter involves a concurrent stressor, such as significant manipulation plus the maintenance of fish out of its aquatic environment. As observed in the study by Franco-Martinez et al. (2022), where an aerial exposure experiment on trout was performed, cortisol levels were much higher in percentage than in the present case, in which hypoxia was achieved by reducing the levels of oxygen from the water by displacement with nitrogen. It should be noted that the physiological stress response is effectively and quickly detected as plasma cortisol increases as early as 3 min post-stress, although the maximum peak is detected after approximately 1 h post-stress (Gesto et al., 2013; Gesto et al., 2015). Fish that have suffered different types of stress (manipulation and hypoxia shocks) show a progressive decrease in glucose levels since it is used as a substrate for glycolytic activity, leading to the release of cell energy (Dunn and Hochachka, 1986). In the present work, this is observed neither in the case of the AC group, which displays a return to initial levels 24 h, nor in H1, which did not display any significant variations in glucose levels throughout the sampling points. Furthermore, in most cases, acute stress induces an increase in plasma glucose, in part because of the energetic needs derived from the stress situation and the mediating effects of cortisol and catecholamines (Abdel-Tawwab et al., 2019). In addition, the absence of significant differences between the different groups at 1 h indicates that the animals have the capacity to replenish their reserves during the 48 h recovery period between different shocks. Moreover, lactate, the product generated by the glycolytic activity (Dunn and Hochachka, 1986), displays significant increases when facing a situation of hypoxia to help maintain cellular energy balance (Dunn and Hochachka, 1986; Richards, 2011), as observed in the present study. This happens because oxygen-independent energetic mechanisms are needed since oxygen-dependent mechanisms are 15 times lower under hypoxic conditions (Richards, 2011). In this sense, the group subjected to one hypoxia is the one with the highest lactate values, so it would be the group that activated anaerobic metabolism the most. It is important to highlight the fact that the group subjected to three exposures and manipulations did not display significant increases in lactate levels, which, again, suggests habituation in the same way as occurs in the case of hematological parameters. This habituation may be facilitated by the mildness of the stressor, which was applied only for short periods of time (Tort et al., 2001).

## 5 Conclusion

The overall results show tolerance and a capacity for a certain degree of habituation in an oxygen-sensitive species such as *O. mykiss* as a response to repeated manipulation and hypoxia. Initial differences are observed in hematological parameters between treated groups and the control, although these differences are overall minimized after three shocks. This return to absolute control levels suggests habituation of some sort, regarding both the correct functioning of red blood cells in transporting and supplying oxygen and white blood cells in host immunity and cell repair. Although cortisol measurements did not return to absolute control levels, they did display a decreasing trend, indicating that, in the long term, fish might be able to recover basal levels, provided that the stressors hypoxia and manipulation maintain the same characteristics. The hypothesis of habituation is further supported by the trends observed in glucose and lactate levels, which display significant alterations associated with changes in aerobic and anaerobic metabolism but return to absolute control levels after the third shock and manipulation. As a general conclusion, it can be stated that rainbow trout are capable of habituating to both these temporary hypoxia episodes and manipulation events without having significant negative consequences at the functional level. However, to firmly affirm this, it would be necessary to analyze how gene and protein expression are modulated since it may help to understand basic molecular and cellular changes associated with the tolerance of hypoxia and manipulation.

## Data Availability

The raw data supporting the conclusion of this article will be made available by the authors, without undue reservation.
